# Normative Values of Peripapillary Retinal Nerve Fiber Layer Thickness in a Middle Eastern Population

**DOI:** 10.1155/2018/7238464

**Published:** 2018-09-17

**Authors:** Mouna M. Al-Sa'ad, Amjad T. Shatarat, Justin Z. Amarin, Darwish H. Badran

**Affiliations:** ^1^Department of Special Surgery, School of Medicine, The University of Jordan, Queen Rania Al-Abdullah Street, Amman 11942, Jordan; ^2^Department of Anatomy and Histology, School of Medicine, The University of Jordan, Queen Rania Al-Abdullah Street, Amman 11942, Jordan; ^3^School of Medicine, The University of Jordan, Queen Rania Al-Abdullah Street, Amman 11942, Jordan

## Abstract

**Purpose:**

Peripapillary retinal nerve fiber layer (pRNFL) thickness is subject to high variability. Normative values of pRNFL thickness remain undocumented in the Middle East. The aim of our study is to assess the normative values of pRNFL thickness in a Middle Eastern population.

**Methods:**

A retrospective chart review of 74 patients was conducted. Outpatients who had presented to the ophthalmology clinic at the Jordan University Hospital between January 2016 and July 2018 were consecutively sampled. Measurements had been recorded using Fourier-domain optical coherence tomography. Multivariable regression models were developed to generate predicted normative values with adjustments to candidate confounders.

**Results:**

The mean global pRNFL thickness was 99 ± 11 *μ*m. The mean quadrantic pRNFL thickness increased from the nasal quadrant (75 ± 16 *μ*m) to the temporal (82 ± 20 *μ*m), superior (114 ± 20 *μ*m), and inferior (125 ± 20 *μ*m) quadrants. Gender and eye sidedness did not contribute to the variability in pRNFL thickness. The relationship between aging and pRNFL thinning is independent of diabetes mellitus type 2 and systemic hypertension. Both systemic conditions significantly predicted pRNFL changes despite negative fundoscopic findings.

**Conclusions:**

Our set of predicted normative data may be used to interpret measurements of pRNFL thickness in Middle Eastern patients. Our findings suggest that systemic conditions with potential ocular manifestations may require consideration in predictive models of pRNFL thickness, even in the absence of gross fundoscopic findings. Normative data from additional Middle Eastern populations are required to appraise our models, which adjust for common clinical confounders.

## 1. Introduction

Glaucoma is an optic neuropathy characterized by excavation of the optic disc, thinning of the peripapillary retinal nerve fiber layer (pRNFL), and a specific pattern of visual field loss. Glaucoma, the leading cause of irreversible vision loss, affects an estimated 66.8 million people worldwide [1]. The diagnosis of glaucoma encompasses a number of clinical observations and measurement techniques [2].

Optic disc excavation and visual field defects are relatively late clinical manifestations of glaucoma. Peripapillary retinal nerve fiber layer thinning precedes these events by a considerable period of time. Therefore, quantitative assessment of pRNFL thickness is useful for the diagnosis of glaucoma in its early stages. Indeed, the measure is highly sensitive, specific, and reproducible [3, 4]. A number of techniques have been used to assess pRNFL thickness, the most notable of which is optical coherence tomography (OCT) [5].

Optical coherence tomography is a noninvasive cross-sectional imaging modality that measures internal structure in biological systems, including ocular structures [5, 6]. The ocular imaging technology is a useful tool in the inventory of the ophthalmologist. For instance, high-resolution *in vivo* imaging of retinal structure is important for the diagnosis of optic neuropathies [7]. The performance of OCT-based imaging is continually improving with further iterations of the technology [8]. Indeed, the latest iterations include Gabor-domain optical coherence microscopy, which may be used to assess the microstructures of the cornea [9]. In the clinic, Fourier-domain OCT is used in standard commercial systems and offers superior sensitivity compared to the conventional time-domain approach [8, 10].

Peripapillary retinal nerve fiber layer thickness is naturally subject to anatomic variation. Therefore, measurements are interpreted against a backdrop of normative reference values. Normative values are readily available, albeit for no more than a select number of ethnic groups [11]. The preceding fact is problematic as normative values may be highly variable between populations. Thus, their documentation in additional populations is necessary [12]. To the best of our knowledge, no study to date has documented the normative values of pRNFL thickness in a Middle Eastern population. The heterogeneity of Middle Eastern populations calls for a series of investigations to determine robust normative values of pRNFL thickness. Herein, we present a preliminary investigation of these values using Fourier-domain OCT.

## 2. Methods

### 2.1. Participants

We conducted a retrospective chart review of outpatients who had presented to the ophthalmology clinic at the Jordan University Hospital between January 2016 and July 2018. Patient data were reviewed on a workstation located in the clinic. All adult patients (≥18 years of age) who underwent complete ophthalmologic assessment and whose data were available were included. Exclusion criteria included any history of retinopathy or optic neuropathy, recent history of ocular surgery (≤1 year), family history of glaucoma, high-degree myopia, use of antiglaucoma agents, poor scan quality, and incomplete binocular data. Institutional Review Board approval was sought and obtained (Jordan University Hospital, Amman, Jordan).

### 2.2. Measures

Peripapillary retinal nerve fiber layer thickness had been measured using a Fourier-domain OCT system (RTVue, Optovue, Inc., Fremont, CA). Measurements had been recorded by the same operator in all cases (MM Al-Sa'ad) using the RNFL3.45 mode. Measurements of pRNFL thickness in five areas (namely, temporal, superior, nasal, inferior, and global) were transcribed. In addition, clinical data (namely, age, gender, history of diabetes mellitus type 2, history of systemic hypertension, and refractive error) were collected.

### 2.3. Data Analysis

Data were entered into the IBM SPSS Statistics Data Editor (IBM Corporation, Armonk, NY). The software package was used to run descriptive statistics, bivariate statistics, and linear regression. The dataset included the complete binocular and clinical profile of each patient. To examine for intraindividual variation in pRNFL thickness, binocular measurements were paired. Difference scores between paired measurements were computed. The scores were examined for outliers and normality using box plots and normal Q–Q plots, respectively. Paired measurements were subsequently compared using the paired‐samples *t*-test. Intraindividual variation was interpreted using a significance level of 0.05.

**Figure 1:**
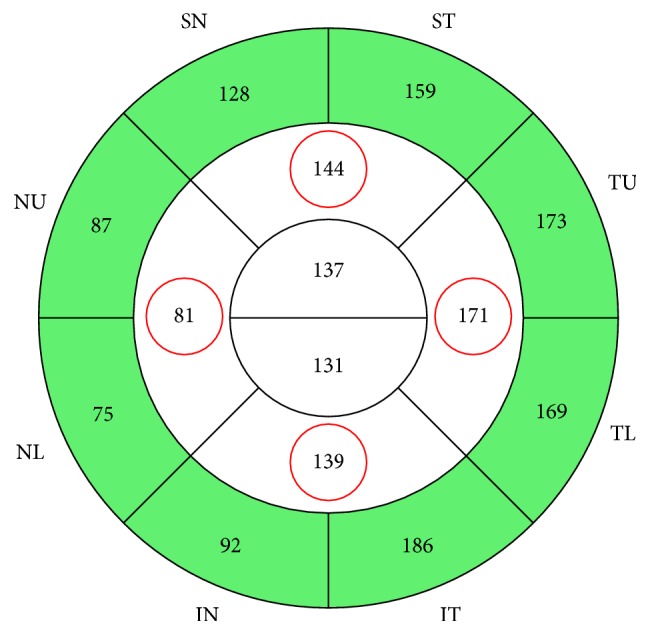
Peripapillary retinal nerve fiber layer (pRNFL) thickness analysis by the manufacturer (Optovue, Inc.). Circled in red are the four quadrantic measurements of pRNFL thickness (*μ*m). The global RNFL thickness is the calculated mean of the four quadrantic measurements.

Multivariable linear regression models were developed to predict the five measurements of pRNFL thickness (Figure 1). Candidate predictors were age, gender, eye sidedness, history of diabetes mellitus type 2, history of systemic hypertension, and refractive error. Bivariate correlations were evaluated by simple linear regression. The significance threshold for model entry was set at an uncorrected value of *P* < 0.2. Predicted normative values were generated from the calculated regression equations using the LMATRIX subcommand. The assumptions underlying linear regression were met. Briefly, plots of studentized residuals and unstandardized predicted values were examined for linearity and homoscedasticity. In addition, partial regression plots were examined for linearity. Multicollinearity was assessed using variance inflation factors. Studentized deleted residuals (>3 or <−3), Cook's *D* (>1), and leverage values (>0.2) were used to detect outliers, highly influential points, and high leverage points, respectively. Normality was assessed using normal P–P plots.

The Benjamini–Hochberg method was used to correct for multiple testing, unless otherwise stated. Numerical data are presented according to the recommendations of Cole [13]. Continuous data are presented as means and standard deviations (separated by a plus-minus sign). Frequencies are presented as absolute and relative values (the latter within parentheses).

## 3. Results

Fourteen patients were excluded for poor scan quality or incomplete binocular data. The final study population comprised 74 patients. One hundred forty-eight eyes entered statistical analysis. Thirty-five patients were male and 39 were female. The patients ranged in age from 18 to 79 years (mean age, 60 ± 12 years). The median spherical equivalent was 0.50 diopters (range, −3.50 to 2.50 diopters). Clinical characteristics of the study population are outlined in Table 1. Paired measurements of pRNFL thickness did not statistically significantly differ in any area (temporal, *P*=1; superior, *P*=0.2; nasal, *P*=0.6; inferior; *P*=0.2; global, *P*=0.2).

The mean global pRNFL thickness in the study population was 99 ± 11 *μ*m. Quadrantic pRNFL thickness measurements are presented in Table 2. The mean quadrantic pRNFL thickness increased from the nasal quadrant to the temporal, superior, and inferior quadrants, in the mentioned order. In 114 eyes (77%), quadrantic measurements of pRNFL thickness did not follow the “ISNT rule” (i.e., inferior > superior > nasal > temporal). In 56 eyes (38%), quadrantic measurements of pRNFL thickness did not follow the “IST rule” (i.e., inferior > superior > temporal).

Gender and eye sidedness did not meet the entry criterion of any model. Refractive error met the entry criterion of the model for inferior pRNFL thickness but did not statistically significantly add to the prediction. In fact, the overall model for inferior pRNFL thickness was not statistically significant (*P*=0.06). In contrast, the remaining models were statistically significant (*P* < 0.001). Age, history of diabetes mellitus type 2, and history of systemic hypertension contributed to the remaining models in variable patterns. Age and history of systemic hypertension generally predicted a decrease in RNFL thickness. However, history of diabetes mellitus type 2 generally predicted an increase in RNFL thickness. Full model results are shown in Table 3.

Predicted normative data are based on the regression equations calculated. The predictions assume a negative history of diabetes mellitus type 2, a negative history of systemic hypertension, and a spherical equivalent of zero. Predicted normative data are presented in Table 4 for one-decade increments in age.

## 4. Discussion

We developed regression models to predict the normative values of pRNFL thickness in a Middle-Eastern population. During model development, we examined pRNFL thickness measurements for sexual dimorphism, binocular asymmetry, age-related changes, and clinical association with diabetes mellitus type 2, systemic hypertension, and refractive error. In addition, we quantified deviations from the “ISNT rule” and the “IST rule” for descriptive purposes. As suggested by previous reports, neither parameter appears to be clinically useful [14, 15].

Ethnic variation in pRNFL thickness has been demonstrated in several studies [11, 12]. Though reports are incongruous, the pRNFL appears to be appreciably thinner in whites compared with Hispanics and Asians [11, 12, 16]. The mean global pRNFL thickness reported herein is highly concordant with data from previous studies on white populations and by extension, the original normative database. In addition, quadrantic measurements of pRNFL thickness in the present study are roughly consistent with previously reported values [12, 16]. It appears, then, that adjustments need not be made to pRNFL thickness measurements in Middle Easterners. However, the Middle Eastern population comprises a heterogeneous admixture of peoples. Therefore, normative data from additional Middle Eastern populations are required to confirm this finding.

Fundoscopic examination of all members in our study population was normal. Interestingly, a history of diabetes mellitus type 2 generally predicted a considerable increase in pRNFL thickness. In contrast, a meta-analysis of 13 studies concluded that pRNFL thickness is significantly decreased in preclinical diabetic retinopathy [17]. However, the ophthalmologic complications of diabetes mellitus type 2 include diabetic retinopathy and diabetic macular edema, and the latter entity has been shown to increase pRNFL thickness [18]. Therefore, the interpretation of pRNFL measurements in the setting of diabetes mellitus type 2 appears impractical. Indeed, Yang et al. have recently suggested a novel index to address the literary muddle [19].

In our study, gender and eye sidedness did not qualify for model entry. In support, previous studies have shown that pRNFL measurements are subject to neither sexual dimorphism nor binocular asymmetry [16, 20]. However, in our study population, age and a history of systemic hypertension generally predicted a considerable decrease in pRNFL thickness. Atherosclerosis in the setting of systemic hypertension appears to be associated with pRNFL thinning [21]. In addition, the relationship between aging and pRNFL thinning appears robust despite the presence of several confounders. In our study, age remained a significant predictor following correction for potential confounders. Indeed, a number of authors have attributed age-related thinning to senescence [12, 16, 20, 22]. The clinical significance of senescence is addressed by our set of predicted normative data.

## 5. Conclusions

In conclusion, our set of predicted normative data may be used to interpret measurements of pRNFL thickness in Middle Eastern patients. Our findings suggest that systemic conditions with potential ocular manifestations may require consideration in predictive models of pRNFL thickness, even in the absence of gross fundoscopic findings. Normative data from additional Middle Eastern populations are required to appraise our models.

## Figures and Tables

**Table 1:** Clinical characteristics of the study population (*N*=74).

Characteristics	Mean or frequency
Age (years)	60 ± 12

*Gender*	
Male	35 (47)
Female	39 (53)

*History of diabetes mellitus type 2*	
Negative	39 (53)
Positive	35 (47)

*History of systemic hypertension*	
Negative	43 (58)
Positive	31 (42)

Continuous data are presented as means and standard deviations (separated by a plus-minus sign). Frequencies are presented as absolute and relative values (the latter within parentheses).

**Table 2:** Mean peripapillary retinal nerve fiber layer (pRNFL) thickness by area (*N*=148 eyes).

Area	Mean pRNFL thickness (*μ*m)
Temporal	82 ± 20
Superior	114 ± 20
Nasal	75 ± 16
Inferior	125 ± 20

Continuous data are presented as means and standard deviations (separated by a plus-minus sign).

**Table 3:** Multivariable regression models of peripapillary retinal nerve fiber layer (RNFL) thickness by area in a Middle Eastern population (*N*=148 eyes).

Variables	*B* value^*∗*^	95% CI	*P* value
*Temporal*			<0.001
Age (years)	−0.35	−0.61 to −0.08	0.01
History of diabetes mellitus type 2	6	−1 to 12	0.09
History of systemic hypertension	11	4 to 17	0.002

*Superior*			<0.001
Age (years)	−0.12	−0.37 to 0.14	0.4
History of diabetes mellitus type 2	11	4 to 17	0.001
History of systemic hypertension	−14	−21 to −8	<0.001

*Nasal*			<0.001
History of diabetes mellitus type 2	10	5 to 15	<0.001
History of systemic hypertension	−12	−17 to −7	<0.001

*Inferior*			0.06
Age (years)	−0.21	−0.47 to 0.06	0.1
Refraction (spherical equivalent power)	−2.5	−5.3 to 0.2	0.07

*Global*			<0.001
Age (years)	−0.16	−0.31 to −0.01	0.04
History of diabetes mellitus type 2	8	4 to 11	<0.001
History of systemic hypertension	−4.2	−8.1 to −0.3	0.04

*B* value, regression coefficient; 95% CI, 95% confidence interval. ^*∗*^*B* values represent the change in the dependent variables (i.e., pRNFL thickness by area) for each unit of change in the independent variables (i.e., predictors).

**Table 4:** Age-adjusted normative values of peripapillary retinal nerve fiber layer thickness by area.

Age (years)	Mean predicted pRNFL thickness (95% confidence interval) in *μ*m				
	Temporal	Superior	Nasal	Inferior	Global
20	89 (78–100)	120 (110–131)	75 (72–79)	134 (122–145)	104 (98–110)
30	85 (77–94)	119 (111–127)	75 (72–79)	132 (123–140)	102 (97–107)
40	82 (76–88)	118 (112–124)	75 (72–79)	129 (123–136)	101 (97–104)
50	79 (74–83)	117 (112–121)	75 (72–79)	127 (123–132)	99 (96–102)
60	75 (71–80)	116 (111–120)	75 (72–79)	125 (122–129)	97 (95–100)
70	72 (66–77)	114 (109–120)	75 (72–79)	123 (119–127)	96 (92–99)
80	68 (61–76)	113 (106–121)	75 (72–79)	121 (115–127)	94 (90–99)

## Data Availability

The data that support the findings of this study are available from the corresponding author upon reasonable request.
